# Electrochemical Disinfection of Simulated Ballast Water Using RuO_2_-TiO_2_/Ti Electrode

**DOI:** 10.3390/ijerph19031835

**Published:** 2022-02-06

**Authors:** Sivasankar Annamalai, Cybelle Concepcion Futalan, Yeonghee Ahn

**Affiliations:** 1 Department of Environmental Engineering, Dong-A University, Busan 49315, Korea; asivasankar87@yahoo.com; 2 Department of Community and Environmental Resource Planning, University of the Philippines, Los Baños 4031, Philippines; cmfutalan@up.edu.ph

**Keywords:** ballast water, *E. coli*, electroactive chlorine species, electrochemical disinfection, RuO_2_-TiO_2_/Ti electrode

## Abstract

The present work investigated the treatment of ballast water via electrochemical disinfection using a RuO_2_-TiO_2_/Ti electrode. Batch tests were conducted with simulated ballast water containing *Escherichia coli* as an indicator organism. The effect of varying NaCl concentrations (1%, 2%, and 3%; *w/v*) and current densities (0.3, 1.0, 2.0, and 3.0 mA/cm^2^) on the inactivation of *E. coli* was examined. Results showed higher disinfection efficiency of *E. coli* was obtained at higher NaCl concentration and current density. Complete inactivation of *E. coli* was attained within 2 and 1 min at 0.3 and 1 mA/cm^2^, respectively, under 3% NaCl concentration. Meanwhile, complete disinfection at 1 and 2% NaCl concentrations was observed in 6 and 2 min, respectively, using a current density of 0.3 mA/cm^2^. The 100% inactivation of *E. coli* was achieved with an energy consumption in the range of 2.8 to 2.9 Wh/m^3^ under the NaCl concentrations at 1 mA/cm^2^ and 1 min of electrolysis time. The complete disinfection attained within 1 min meets the D-2 standard (<250 CFU *E. coli*/100 mL) of ballast water under the International Maritime Organization. The values of energy consumption of the present work are lower than previous reports on the inactivation of *E. coli* from simulated ballast water.

## 1. Introduction

Ballast water is fresh, or seawater held in the ballast tanks of ships [1]. It provides stability and maneuverability to the ships during a voyage. Approximately 10 billion tons of ballast water is discharged annually into the marine environment since ships transport 80% of the globally traded goods and merchandise [1,2]. Ballast tanks were reported to contain a minimum of 7000 various species that were transported worldwide daily according to the International Maritime Organization (IMO) [1,2]. The organisms (e.g., zooplankton, bacteria, dinoflagellates, larvae, etc.) in ballast water were shown to survive during transportation [3,4,5]. The ballast water discharge affects the marine environment, ecology, economy, and human health since ballast water carries invasive species along with pathogens to aquatic organisms and humans [1,2].

IMO adopted the International Convention for the Control and Management of Ships’ Ballast Water and Sediments (BWM Convention) in 2004 to introduce global regulations to control the transfer of potentially invasive species [6]. All ships in international traffic need to manage their ballast water since the BWM Convention entered into force globally. Under the Convention, new ships built on or after 8 September 2017 must meet the D-2 standard while all ships must meet the standard by 8 September 2024. The D-2 standard specifies the maximum concentration of viable organisms allowed to be discharged, including specified indicator microorganisms (e.g., <250 CFU *E. coli*/100 mL; <1 CFU *Vibrio cholera*/100 mL) [2,6].

Various technologies were employed to inactivate and disinfect aquatic organisms and microbes in ballast water [1,2,7,8,9,10]: ultraviolet radiation, biocide application, sonication, filtration, ozone treatment, and chemical method. Currently, these methods have limitations such as toxic by-product formation, large space requirement, high energy consumption, low treatment efficiency, and chemical storage requirement. In recent years, previous reports on electrochemical studies have shown that electrochemical treatment can effectively remove contaminants or pathogens from the environment [11,12,13]. Electrochemical disinfection has gained increasing attention due to its ability to inactivate and remove various living organisms and pathogens from water [13,14]. However, it is an emerging method for ballast water treatment.

Electrochemical disinfection uses suitable electrode materials to create a direct current (DC) electric field that flows the water and inactivates microorganisms. It has numerous advantages such as easy operation and installation, simple instruments, and high treatment efficiency [2,13,14,15]. In addition, electrochemical disinfection has a small energy requirement, which makes it cost-effective and environmentally compatible that could be sustained using fuel cells or solar cells [2,13,14,15,16]. More importantly, electrochemical disinfection can produce oxidizing species in situ that would not require the storage and transport of chemicals [2,14]. Since seawater, which is commonly used as ballast water, naturally contains chloride ions, the production of chlorine species such as hypochlorous acid (HOCl) and hypochlorite (ClO^−^) during electrochemical disinfection is an advantage [2,14]. The inactivation efficiency of the process is attributed to electrochemical chlorination and the presence of reactive oxygen species (e.g., hydrogen peroxide, hydroxyl radicals). The generation of both chlorine species and reactive oxygen species would cause the deterioration of the microbial cell wall and membrane, which leads to cell lysis [13,14,17].

Selecting a proper anodic material is essential in the electrochemical process since it affects the process efficiency and the formation of electroactive chlorine species (e.g., HClO, ClO^−^, Cl_2_) that would be generated when chloride salts are present [13,14,18,19]. Researchers investigated various anode materials for ballast water treatment: boron-doped diamond (BDD), PbO_2_, graphite, platinum (Pt), and metal oxide coated titanium electrodes [13,20,21,22]. When PbO_2_/graphite felt anode was employed to treat the artificial seawater (3% NaCl), the complete inactivation of *E. coli*, *Enterococcus faecalis*, and *Artemia salina* was achieved at 8, 60, and 40 min, respectively, using a current density of 25.3 mA/cm^2^ [21]. Lacasa et al. [22] reported that 100% disinfection of *E. coli* was attained within 5 min using BDD to treat simulated ballast water (3% NaCl). The energy consumption was determined to be 42 Wh/m^3^ at the current density of 12.7 mA/cm^2^.

Dimensionally stable anodes (DSAs) are comprised of a titanium substrate coated with inorganic metal oxides (e.g., SnO_2_, IrO_2_-Sb_2_O_5_-SnO_2_, TiO_2_-RuO_2_, and IrO_2_-RuO_2_) and are known for their resistance to corrosion and superior electrical conductivity [17,23,24]. DSAs have been widely applied for the disinfection of water [15,17,23,24]. When compared to other stable anodes like Pt and BDD, DSAs have higher efficiency in generating chlorine species and better disinfection dosage that allow the process to be operated at a lower voltage range and decreased energy consumption [25,26]. DSAs commonly use conductive metal oxides such as TiO_2_, IrO_2,_ and RuO_2_ as coating film. The combination of metal oxides would develop a synergistic, bifunctional anode that is both electrocatalytic and photocatalytic. The application of RuO_2_ in industries is associated with its excellent conductivity, high electrocatalytic activity, and exceptional stability [27,28,29]. Previous studies mainly used electrodes with the mole ratio of 30:70 for RuO_2_ and TiO_2_ to degrade organic contaminants for wastewater treatment [30]. There are no studies performed on the varying molar ratio of RuO_2_ that could further improve the electrocatalytic activity of the anode material and enhance the disinfection efficiency for ballast water treatment.

Studies on better methods for ballast water treatment are very much necessary before the implementation of D-2 compliance in all the ships. In this regard, this study prepared mixed metal oxide (RuO_2_-TiO_2_/Ti; 45 mol % RuO_2_ + 55 mol % TiO_2_) as the anode material and investigated its performance to disinfect simulated ballast water containing *E. coli* in the electrochemical process. The prepared RuO_2_-TiO_2_/Ti anode was characterized using a scanning electron microscope (SEM) and energy-dispersive x-ray spectroscopy (EDS). The inactivation efficiency of *E. coli* was investigated under varying process parameters such as current density and NaCl concentration. The energy consumption and decay of *E. coli* under different salinity were also determined.

## 2. Materials and Methods

### 2.1. Preparation and Characterization of Electrode

The mixed metal oxide of RuO_2_-TiO_2_/Ti expanded mesh anode was prepared by pyrolysis of titanium tetrachloride (TiCl_4_) and ruthenium trichloride (RuCl_3_) on titanium (Ti) substrate. To attain a rough surface, the expanded Ti mesh was sandblasted and etched with 10% HCl for 3 min at 80 °C. Then, the chemically-etched Ti expanded mesh was washed with distilled water and air-dried. After which, the dissolution of RuCl_3_ and TiCl_4_ (concentration of 45:55 mol %) in isopropanol was carried out. The solution mixture was brushed on the surface of the pretreated Ti expanded mesh. Then, the electrode was burnt at 400 °C for 10 min at O_2_ atmosphere and was air-dried for 10 min. This step was repeated until a thickness of 6 μm coating layer was obtained [31]. Finally, the final firing was done at 420 °C for 1 h. The electrode surface of the RuO_2_-TiO_2_/Ti expanded mesh anode was analyzed by SEM (Hitachi SU-70, Hitachi High-Technologies, Tokyo, Japan) and EDS (INCA, Oxford Instruments, Oxfordshire, UK).

### 2.2. Bacterial Strains and Growth Conditions

*E. coli* CN13 was employed as an indicator organism in this study. A single colony of the strain grown on a Luria Bertani (LB) agar plate was inoculated into 500 mL of sterile LB broth and cultured at 37 °C in a shaking incubator at 150 rpm for 48 h. The cells were harvested by centrifugation (Model Mega 17R; Hanil, Daejeon, Korea) for 10 min at 10,000 rpm and 20 °C. The harvested cells were washed and resuspended in varying NaCl solutions (1, 2, and 3%; *w*/*v*) that were used as the background electrolyte. NaCl solutions were prepared with deionized water [17,21]. The bacterial suspensions (~1 × 10^7^ CFU/mL) in the NaCl solutions were used for the disinfection tests.

### 2.3. Set-Up of the Electrochemical Reactor

The present work employed an electrochemical reactor similar to the set-up described previously [17,21]. Batch electrolysis experiments were carried out in a 300 mL beaker (undivided cell) with a working volume of 250 mL. The simulated ballast water was prepared as described for NaCl solutions [17,21]. The prepared RuO_2_-TiO_2_/Ti expanded mesh served as the anode, while Ti expanded mesh was used as the cathode. An inter-electrode distance of 1 cm was maintained between the anode and cathode, which were placed parallel to each other.

### 2.4. Disinfection Experiments

Before the start of each experiment, sonication of the electrode was performed for 10 min in deionized water to eliminate residual contamination on the electrode surface. The electrochemical disinfection experiments were carried out under constant bacterial concentration (~1 × 10^7^ CFU/mL) and varying current densities (0, 0.3, 1, 2, and 3 mA/cm^2^) in a galvanostatic mode with a regulated DC power source (Model OPE-303QI; ODA Technologies, Incheon, Korea). The electrolyte solution was constantly stirred at 150 rpm using a magnetic stirrer. The samples were collected periodically and the potential was measured. The standard analytical procedure (Iodometric method) was employed to analyze the electroactive chlorine species [21]. All experiments were conducted in triplicate.

### 2.5. Bacterial Sample Analysis

The plate counting method was used to measure *E. coli* concentration in the ballast water before and after each experiment. Samples were collected at specific time intervals: 0.5, 1, 2, 3, 4, 5, 10, 20, and 30 min. During sample collection and handling, an equal volume of 1 N Na_2_S_2_O_3_ was added immediately in the samples to remove residual free chlorine and prevent the death of microbes [32]. Then, the samples were serially diluted for microbial analysis. Each dilution of 100 µL was spread onto an LB agar plate and incubated at 35 °C for 14 h. The colonies grown on the plates were counted.

## 3. Results and Discussion

### 3.1. Characterization and Surface Morphology Analysis

**Figure 1:**
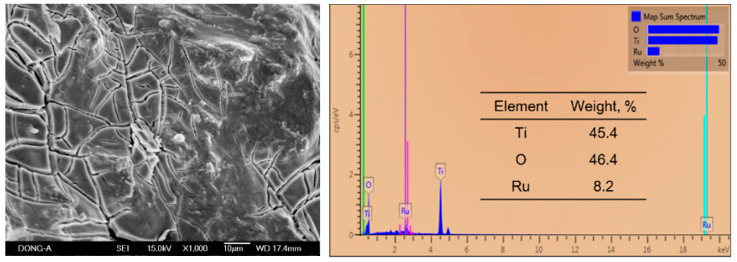
SEM (**left**) and EDS (**right**) surface mapping analysis of RuO_2_-TiO_2_/Ti electrode employed in this study.

The surface morphology of the prepared mixed metal oxide electrode (RuO_2_-TiO_2_/Ti) clearly showed a rough surface morphology with several muddy cracks on the coated electrode (Figure 1). The presence of the cracks increases the surface area of the anode and increases the number of active sites exposed [30]. The EDS analysis verified the presence of Ti and Ru (Figure 1). The phase composition was calculated using element-to-stoichiometric oxide conversion factors. Results show that the electrode surface was comprised of ~75% of Ti and 25% of Ru. During the electrolysis process, the anodic potential was determined to be within the ranges of 1.11–1.34 V, 1.19–1.38 V, and 1.2–1.4 V for 1%, 2%, and 3% NaCl, respectively. The result of potential measurements suggested that RuO_2_–TiO_2_/Ti anode could form electroactive chlorine species [33].

### 3.2. Effect of Current Density

**Figure 2:**
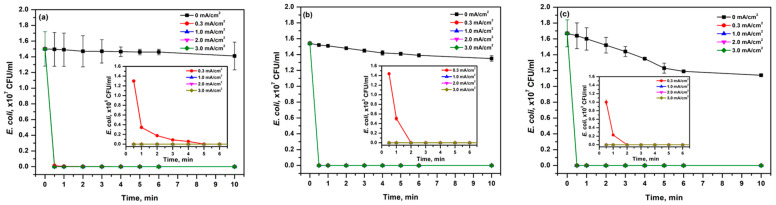
Effect of current density on *E. coli* inactivation at (**a**) 1%, (**b**) 2%, and (**c**) 3% NaCl during electrochemical disinfection treatment. Inner graphs show the inactivation during the initial 6 min. Data are given as mean ± standard deviation (*n* = 3).

Figure 2 shows the effect of current density as a function of time on *E. coli* disinfection. Increasing the current density from 0.3 to 3.0 mA/cm^2^ showed better inactivation efficiency of *E. coli*. In addition, longer contact time also results in better inactivation of *E. coli*. Complete inactivation of *E. coli* was achieved within 1 min when the current density was increased from 0.3 to 1 mA/cm^2^ in all systems. *E. coli* concentration in 1% NaCl solution decreased gradually within 4 min with a current density of 0.3 mA/cm^2^ and showed complete disinfection at 6 min of electrolysis (Figure 2a). Meanwhile, a shorter time (2 min) was needed to attain complete disinfection for 2% and 3% NaCl solutions with the same current density (Figure 2b,c).

Faster reduction of *E. coli* concentration was observed with higher current density due to increased production of electroactive chlorine species and presence of a high number of oxidizing species as well as higher residual chlorine concentration, which can easily disinfect available microbes. In addition, a higher amount of electrical charge will run through the electrolyte with the application of higher current density that could lead to quicker disinfection rates [21]. Similar results were observed by Ghasemian et al. [17] where the disinfection of *E. coli* D21 using Sb-doped Sn_80%_-W_20%_-oxide anode at different current densities was investigated. The complete disinfection of *E. coli* D21 in 0.1M NaCl was obtained at 60, 5, and 2 min with the current density of 1, 2, and 4 mA/cm^2^, respectively. In this study, lower values of current density such as 0.3 mA/cm^2^ were sufficient for the effective inactivation of *E. coli* in simulated ballast water. The utilization of lower current density may avoid higher energy consumption for the disinfection process. The complete inactivation of *E. coli* was attained with 2 min contact time, 0.3 mA/cm^2,^ and 2% NaCl concentration, which meets the IMO D-2 standard (<250 CFU/100 mL).

Bacterial decay occurred with respect to time even without applying current in the reactor due to a low nutrient and/or a highly saline environment. The bacterial concentration in the control (0 mA/cm^2^ applied) slowly decreased from 1.50 × 10^7^ to 1.41 × 10^7^, 1.54 × 10^7^ to 1.35 × 10^7^ and ~1.67 × 10^7^ to 1.14 × 10^7^ CFU/mL for 1%, 2%, and 3% NaCl, respectively.

### 3.3. Kinetics of E. coli Inactivation

**Figure 3:**
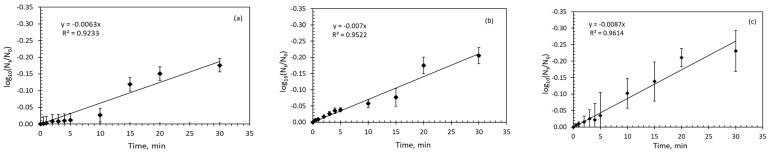
First-order kinetics for the inactivation of *E. coli* at (**a**) 1%, (**b**) 2%, and (**c**) 3% NaCl without applying current. Data are given as mean ± standard deviation (*n* = 3).

The inactivation kinetics of *E. coli* under varying current density and NaCl concentration was investigated. Chick’s law is used to determine the kinetic constants for the inactivation of microorganisms as provided in Equation (1) [34].

(1) $$log_{10}\left(\frac{N_{t}}{N_{0}}\right)=-Kt$$

where *N_t_* is the concentration of *E. coli* at time *t*, *N*_0_ is the initial concentration of *E. coli*; *K* is death rate (min^−1^), and *t* is time (min). Figure 3 shows that experimental data points are in good agreement with the regression lines, which indicates that the first-order kinetic equation is a good fit in describing the inactivation of *E. coli*. The kinetic equations and regression coefficients are presented in Table 1. Results show that the high values of the coefficient of determination (R^2^ ≥ 0.92) were obtained when no current density was applied, while values of R^2^ ≥ 0.79 were attained for a current density of 0.3 mA/cm^2^. The high R^2^ values imply that first-order kinetics would best describe the inactivation of *E. coli* for both systems. This indicates that the disinfection of *E. coli* is controlled by mass transfer or generated reagents (e.g., electroactive chlorine species, oxidizing species) [22]. The values of the kinetic constant (*K*) were determined to be in the ranges of 0.0063–0.0087 min^−1^ for 0 mA/cm^2^ and 0.7076–4.8286 min^−1^ for 0.3 mA/cm^2^. Higher *K* values obtained at higher salinity and higher current density indicated better inactivation efficiency of *E. coli*. Lacasa et al. [22] reported similar findings where the mortality rate of *E. coli* increased with increasing current density and salinity.

### 3.4. Effect of NaCl Concentration

**Figure 4:**
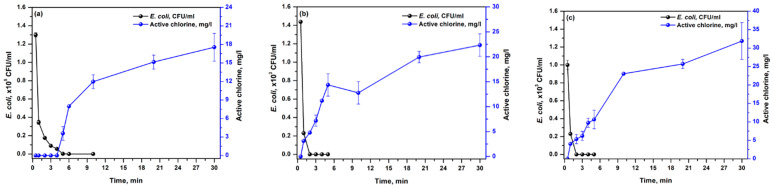
Inactivation performance and concentration of active chlorine during electrochemical disinfection at 0.3 mA/cm^2^. *E. coli* was contained in simulated ballast water containing (**a**) 1%, (**b**) 2%, and (**c**) 3% NaCl, respectively. Data are given as mean ± standard deviation (*n* = 3).

Figure 4 shows the effect of NaCl concentration on *E. coli* disinfection and the production of electroactive chlorine substances at a current density of 0.3 mA/cm^2^. It was observed that increasing the NaCl concentration would lead to better inactivation efficiency of *E. coli*. Results show that the *E. coli* concentration decreased from ~1.50 × 10^7^ to 0.0037 × 10^7^, ~1.54 × 10^7^ to 0.00018 × 10^7^, and ~1.67 × 10^7^ to 0.0001 × 10^7^ CFU/mL for 1%, 2%, and 3% NaCl, respectively, at 1 min of electrolysis. A shorter contact time of 2 min was needed to obtain complete disinfection for both 2% and 3% NaCl while a longer contact time of 6 min was required for 1% NaCl. Increasing the NaCl concentration from 1% to 3% indicates that a higher amount of electroactive chlorine species would be generated; thus, a shorter contact time is required for the complete inactivation of *E. coli.*

As the NaCl concentration was increased from 1 to 3% at a fixed current density of 0.3 mA/cm^2^, a corresponding increase in the electroactive chlorine species concentration was also observed (Figure 4). For the first 4 min, no electroactive chlorine species were observed in 1% NaCl (Figure 4a). After 5 min of electrolysis, 3 mg/L of electroactive chlorine species were measured. Similarly, electroactive chlorine species were not detected at 0.5 min of electrolysis in 2 and 3% NaCl system (Figure 4b,c). This indicates that the electroactive chlorine species were almost completely utilized for the disinfection of *E. coli* during the start of the process. After complete disinfection was attained at 2 min for 2% and 3% NaCl and 6 min for 1% NaCl, the concentration of electroactive chlorine was observed to increase gradually.

Table 2 shows the concentration of electroactive chlorine species under varying current densities and NaCl concentrations after 30 min contact time. The available electroactive chlorine species generated with 0.3 mA/cm^2^ was determined to be 17.5, 22.3, and 31.9 mg/L at 1%, 2%, and 3% NaCl, respectively. A high NaCl concentration and high current density generated an increased amount of electroactive chlorine species. The highest value of electroactive chlorine species (262.35 ± 41.36 mg/L) was attained at 3% NaCl and 3.0 mA/cm^2^. The previous study also showed similar results where complete disinfection of *E. coli* in simulated ballast water at 3% NaCl was attained at 8 min of electrolysis using PbO_2_/graphite electrode at 25.3 mA/cm^2^ [21]. Meanwhile, the present study obtained 100% inactivation of *E. coli* after 2 min of electrolysis using RuO_2_-TiO_2_/Ti anode at 0.3 mA/cm^2^.

### 3.5. Mechanism of E. coli Inactivation

The present study used RuO_2_-TiO_2_/Ti and Ti mesh as anode and cathode, respectively. As the current is applied, electro-migration occurs where the movement of the chloride ions towards the anode occurs. The production of a chlorine molecule (Cl_2_) takes place due to the interaction of Cl^-^ with the electrode surface via direct oxidation (Equation (2)). Simultaneously, OH^−^ ions are formed due to the reduction of water molecules at the cathode (Equation (3)). At the anode, the dissolution of the Cl_2_ gas in water would lead to the formation of electroactive chlorine species such as HOCl and ClO^−^ (Equations (4) and (5)) that are well-known for their bactericidal effects on microorganisms.

(2) $${\mathrm{Cl}}^{-}\;+\;{\mathrm{Cl}}^{-}\;\to \;{\mathrm{Cl}}_{2}\;+\;{2e}^{-}\;\;\;\;\;\;\;\;\;(\mathrm{Anodic}\;\mathrm{reaction})$$

(3) $${2H}_{2}O\;+\;{2e}^{-}\;\to \;H_{2}\;+\;{2\mathrm{OH}}^{-}\;\;\;\;(\mathrm{Cathodic}\;\mathrm{reaction})$$

(4) $${\mathrm{Cl}}_{2}\;+\;H_{2}O\;\;\to \;H^{+}\;+\;{\mathrm{Cl}}^{-}\;{+}^{\;}\mathrm{HOCl}\;\;\;\;\;\;(\mathrm{pH}\;<\;7)$$

(5) $${\mathrm{Cl}}_{2}\;+\;{O\;H}^{-}\;\to \;{\mathrm{ClO}}^{-}\;+H^{+}\;+\;{\mathrm{Cl}}^{-}$$

(6) $$\mathrm{HOCl}\;\;\to \;H^{+}\;+\;{\mathrm{ClO}}^{-}\;\;\;\;\;\;\;\;\;(\mathrm{pH}\;>\;7)$$

Another mechanism of inactivation is via electronic adsorption of microorganisms that are negatively charged onto the anodic surface of the electrode. Cell lysis is caused by the direct transfer of electrons after *E. coli* cells have attached to the anodic surface [35]. Previous reports have shown that the essential mechanism for the inactivation of microorganisms involves electrolyzed chlorine and hydroxyl radicals [36,37]. Particularly, studies on electrochemical disinfection using DSAs have shown that the vital inactivation mechanism is via chlorination in wastewater and surface waters [22,38].

**Figure 5:**
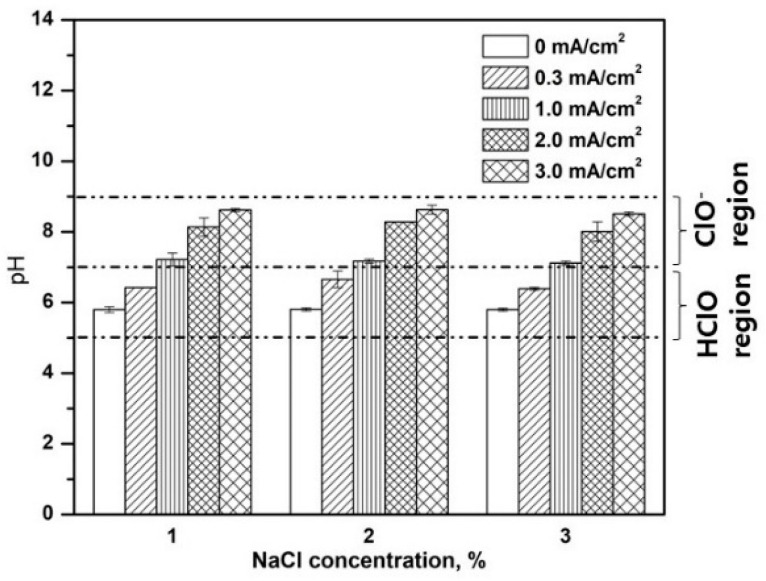
pH change of the electrochemical system after 30 min of electrochemical disinfection at various NaCl concentrations and current densities. Data are given as mean ± standard deviation (*n* = 3).

The pH of the electrochemical system was monitored after 30 min of the electrochemical disinfection process at varying current densities and NaCl concentrations. The control system (0 mA/cm^2^) used in this study showed a constant pH of 5.8 ± 0.05 (Figure 5). The pH of the system seems unaffected under different NaCl concentrations. The pH of the system was determined to be approximately 6.4, 7.2, 8.4, and 9.0, with current density at 0.3, 1, 2, and 3 mA/cm^2^, respectively. When low current density (0.3 and 1 mA/cm^2^) is applied, the pH of the system is neutral (~pH 7.0). Meanwhile, a more basic pH of the solution was observed at a higher current density (2 and 3 mA/cm^2^) due to the formation of hydroxide ions (OH^-^) at the cathode caused by a reduction reaction. Basic conditions such as pH > 8.0 would result in the preferential adsorption of OH^-^ ions onto the anode surface, which in turn, would cause low chlorine generation and decreased disinfection efficiency [2,39]. Most importantly, the stability of the electroactive chlorine species (e.g., HOCl, ClO^−^) is highly dependent on the pH of the electrolyte. Chlorine species such as HOCl and ClO^−^ are more stable in acidic and basic conditions, respectively. Acidic conditions tend to produce a higher concentration of HOCl while basic conditions would lead to the formation of ClO^−^. Meanwhile, both ClO^−^ and HOCl have been determined to be present in the solution with neutral pH. Therefore, acidic and neutral conditions are considered to favor improved inactivation efficiency. In the present work, the application of low current densities (0.3 and 1 mA/cm^2^) with acidic to neutral pH implies the system is conducive to the formation of both ClO^-^ and HOCl. Overall, the measured pH for all systems was less than 9, indicating higher production of electroactive chloride species to be utilized in the disinfection of *E. coli*.

### 3.6. Energy Consumption and Cost Analysis

In general, improved bacterial disinfection is associated with the application of high current density that would result in increased energy consumption. The cost-effectiveness of the electrochemical disinfection process using RuO_2_-TiO_2_/Ti in the disinfection of *E. coli* was examined by determining the energy consumption. The energy consumption (*E*, Wh/m^3^) is computed using Equation (7) [18,30]:

(7) $$Energy\;consumption\;\left(E\right)=\frac{1}{V_{s}}\int V\;I\;dt$$

where *V_s_* is the volume of sample processed, *V* is the voltage difference between the electrodes and *I* is the electric current.

The energy input to obtain complete bacterial disinfection was determined to be in the range of 2.9–10.4 Wh/m^3^ (1% NaCl), 2.8–8.7 Wh/m^3^ (2% NaCl) and 2.8–8.0 Wh/m^3^ (3% NaCl) under various current densities (0.3–3 mA/cm^2^). The lowest power consumption of 2.8 Wh/m^3^ using RuO_2_-TiO_2_/Ti anode was able to attain 100% bacterial disinfection of 1 × 10^7^ CFU/mL *E. coli* under the following conditions: 1 mA/cm^2^ of current density, 2% to 3% NaCl concentrations, and 1 min of electrolysis time. The values of energy consumption obtained in the present work are lower when compared to previous reports. Heim et al. [40] reported that complete disinfection of *E. coli* (10^7^–10^8^ CFU/mL) in municipal wastewater was achieved with an energy consumption of 250 Wh/m^3^ under different NaCl concentrations (2% and 3%) using BDD. About 88 Wh/m^3^ of energy input was required in the study of Lacasa et al. [22] to obtain complete inactivation of *E. coli* (10^6^–10^7^ CFU/mL) using BBD at 3% (*w*/*v*) NaCl. The present work demonstrated that 1 mA/cm^2^ of current is sufficient to attain 100% disinfection of *E. coli* in the simulated ballast water.

## 4. Conclusions

In the present study, RuO_2_-TiO_2_/Ti anode (45 mol % RuO_2_ + 55 mol %TiO_2_) was developed and used in the electrochemical disinfection of *E. coli* from simulated ballast water. Results show that increasing the NaCl concentration (0–3%; *w*/*v*) and current density (0.3–3.0 mA/cm^2^) would cause better inactivation efficiency of *E. coli* under shorter electrolysis time due to the production of higher concentration of electroactive chlorine species and oxidizing species in the system. The concentration of the total electroactive chlorine species present during electrochemical disinfection was determined to be within the range of 17.50–262.35 mg/L under varying current densities and NaCl concentrations. Complete disinfection of *E. coli* was achieved at a shorter contact time of 2 min for both 2% and 3% NaCl while a longer contact time of 6 min was needed for 1% NaCl at a current density of 0.3 mA/cm^2^. The energy input required to attain complete disinfection of *E. coli* was determined to be 2.9 ± 0.07, 2.8 ± 0.,07 and 2.8 ± 0.10 Wh/m^3^ for 1%, 2%, and 3% NaCl, respectively, using a current density of 1 mA/cm^2^ and 1 min of electrolysis time. The energy consumption values in the present work are lower when compared to those in previous reports in the inactivation of *E. coli* from simulated ballast water. In the present study, 100% disinfection of *E. coli* was attained in 1 min at 1 mA/cm^2^ that meets the IMO D-2 standard (<250 CFU *E. coli*/100 mL) of ballast water.

## Figures and Tables

**Table 1:** First-order kinetics of *E. coli* decay at various NaCl concentrations.

Current Density, mA/cm^2^	Kinetics	NaCl conc., % (*w*/*v*)		
		1	2	3
0	First-order	$\begin{array}{l} \log_{10}\left(\frac{N_{t}}{N_{0}}\right)\;\\ =\;0.0063\;\times \;t \end{array}$	$\begin{array}{l} \log_{10}\left(\frac{N_{t}}{N_{0}}\right)\;\\ =\;0.0070\;\times \;t \end{array}$	$\begin{array}{l} \log_{10}\left(\frac{N_{t}}{N_{0}}\right)\;\\ =\;0.0087\;\times \;t \end{array}$
	R^2^ *	0.92	0.95	0.96
0.3	First-order	$\begin{array}{l} \log_{10}\left(\frac{N_{t}}{N_{0}}\right)\;\\ =\;0.7076\;\times \;t \end{array}$	$\begin{array}{l} \log_{10}\left(\frac{N_{t}}{N_{0}}\right)\;\\ =\;4.4699\;\times \;t \end{array}$	$\begin{array}{l} \log_{10}\left(\frac{N_{t}}{N_{0}}\right)\;\\ =\;4.8286\;\times \;t \end{array}$
	R^2^	0.79	0.82	0.84

* Regression coefficient.

**Table 2:** Formation of electroactive chlorine species at 30 min of electrolysis.

NaCl, % (*w*/*v*)	Current Density, mA/cm^2^	Electroactive Chlorine Species, mg/L
1	0.3	17.50 ± 2.25 *
	1	55.83 ± 1.12
	2	156.34 ± 10.15
	3	207.39 ± 13.15
2	0.3	22.30 ± 2.25
	1	60.62 ± 7.89
	2	169.11 ± 14.66
	3	252.06 ± 1.12
3	0.3	31.90 ± 5.01
	1	79.76 ± 9.02
	2	162.72 ± 15.79
	3	262.35 ± 41.36

* Data are given as mean ± standard deviation (*n* = 3).

## Data Availability

Data are within the article.
